# Integrative optical genome mapping and long-read sequencing dissect a pathogenic complex structural variant in a multigenerational Chinese retinitis pigmentosa pedigree

**DOI:** 10.1016/j.gendis.2025.102008

**Published:** 2025-12-23

**Authors:** Yuqiao Ju, Cong Duan, Kaixin Chen, Qing Chang, Yuan Zong, Fengjuan Gao, Ge-zhi Xu

**Affiliations:** a Eye Institute and Department of Ophthalmology, Eye & ENT Hospital, Fudan University, Shanghai 200031, China; b Shanghai Key Laboratory of Visual Impairment and Restoration, Shanghai 200031, China; c NHC Key Laboratory of Myopia and Related Eye Diseases, Key Laboratory of Myopia and Related Eye Diseases, Chinese Academy of Medical Sciences, Shanghai 200031, China; d Yunnan Eye Institute & Key Laboratory of Yunnan Province, Yunnan Eye Disease Clinical Medical Center, Affiliated Hospital of Yunnan University, Kunming, Yunnan 650021, China

Retinitis pigmentosa (RP), characterized by the progressive degeneration of photoreceptor cells, giving rise to night blindness, visual field constriction, and subsequent central-vision loss, is associated with more than 100 genes.^1^ The advent of next-generation sequencing has revolutionized genetic diagnostics, enabling the highly accurate identification of single-nucleotide variants.^2^ However, the overall genetic diagnosis rate for RP remains suboptimal as the positive rate of genetic testing only ranges from 50% to 70%.^3^ Recent studies have found that among cases with negative next-generation sequencing results, a subset may be attributed to complex structural variants (SVs), as next-generation sequencing relies on hybridization-capture technology with short read lengths and has limitations in detecting large-scale SVs.^2^ To tackle these challenges, advanced methods such as long-read sequencing (Oxford Nanopore Technologies, ONT) and optical genome mapping (OGM) have emerged as potent tools for resolving complex genomic rearrangements.^3^^,^^4^ These methods provide longer read lengths, haplotype resolution, and the capability to detect inversions or translocations, rendering them invaluable for studying SVs in various inherited disorders.

Here, we investigated a large multigenerational pedigree with potential autosomal-dominant RP and employed whole-genome sequencing (WGS), ONT, and OGM to dissect the underlying variants. The detailed methods are presented in the supplementary data.

The proband, a 60-year-old female, first presented to the clinic with a six-month history of progressive bilateral visual field constriction and reported childhood-onset nyctalopia. She reported no systemic comorbidities potentially linked to her ocular manifestations. Her daughter also complained of night blindness. Comprehensive ophthalmological examinations revealed that the phenotype of the fundus in both mother (Fig. 1A) and daughter (Fig. 1B) was consistent with typical RP, with widespread osteocyte-like pigment clumping, extensive atrophy, and macular outer retinal layer loss. Family history revealed autosomal-dominant inheritance: the proband's mother, three siblings, and multiple relatives reported comparable symptoms. Pedigree analysis confirmed a large multigenerational RP pedigree (Fig. 1C), with the proband designated as F_II-6_.

**Figure 1:**
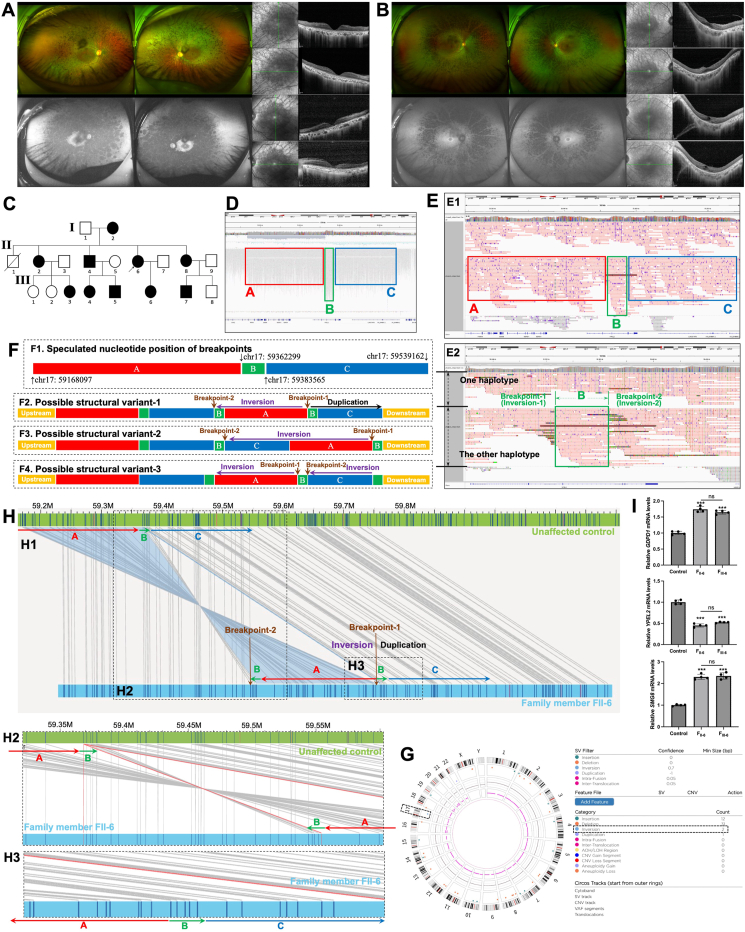
Integrative advanced methods of genetic testing unveil a pathogenic complex structural variant (SV) in a large Chinese pedigree with retinitis pigmentosa (RP). **(A)** Fundus photography of the proband demonstrates widespread osteocyte-like pigment clumping and attenuated retinal vasculature. Fundus autofluorescence imaging shows extensive atrophy with residual posterior hyperfluorescent areas bilaterally. Spectral-domain optical coherence tomography (SD-OCT) indicates macular outer retinal layer loss, with the left eye complicated by an epiretinal membrane. **(B)** Fundus photography and SD-OCT of the daughter exhibit similar but milder phenotypic features than the proband (her mother): fundus pigmentary changes, epiretinal membranes, and mild cystoid macular edema. **(C)** Pedigree analysis of the multigenerational RP family. The proband is designated as F_II-6_. **(D)** Whole-genome sequencing (WGS) data of F_II-6_ presented via Integrative Genomics Viewer (IGV). Chromosome positions: Segment A, red, chr17: 59168097–59362996 (length: 195 Kbp), with 3 copies; Segment B, green, chr17: 59362996–59383562 (length: 21 Kbp), with 4 copies; Segment C, blue, chr17: 59383562–59539137 (length: 156 Kbp), with 3 copies. Breakpoints were mapped to chr17: 59168097 and chr17: 59539137 (hg38). **(E)** Long-read sequencing (Oxford Nanopore Technologies, ONT) data of F_II-6_ presented via IGV. **(E1)** IGV visualization of ONT data (wide view). Two haplotypes are separated in the IGV using long-read length comparisons, confirming that the SV resided on a single haplotype. Copy number analysis reveals segment-specific dosage patterns: 2 copies for segments A and C, and 3 copies for segment B, consistent with WGS findings. **(E2)** IGV of ONT data, a narrow range of views. Red-highlight read, the only read spans the whole segment B. Segment B exhibits several reads on both sides that fail to align with the transcript (displaying mixed colors rather than orange), suggesting the presence of inversions. Breakpoint mapping identifies two inversions flanking segment B: inversion-1 at chr17:59168097–59362996 (hg38) and inversion-2 at chr17:59383565–59539162 (hg38). **(F)** The exact architecture analysis based on WGS and ONT. **(F1)** Mapping of chromosome positions and breakpoints of the potential SV. **(F2–4)** Plausible configurations of the potential SV. Variant-1∼3 indicate different possibilities, including nested duplication-inversion, tandem triplication with interspersed inversions, and complex insertion. **(G)** The *de novo* assembly map of optical genome mapping (OGM). The results indicated by the dotted boxes reveal two inversions. **(H)** The aberrant label pattern of OGM. **(H1)** Full field of view of the target segment. The green band above represents the unaffected control, while the blue band below represents the RP patient (F_II-6_). DNA methyltransferase sites (marked blue) are connected by a grey line between the unaffected control and F_II-6_; DNA methyltransferase sites of F_II-6_ within the inversion display the reverse order of the unaffected control, whereas DNA methyltransferase sites within duplications align with the unaffected control. **(H2)** Magnified view of breakpoint junctions (Breakpoint-2). **(H3)** Magnified view of breakpoint junctions (Breakpoint-1). **(I)** Relative *GDPD1*, *YPEL2*, and *SMG8* mRNA expression levels in the peripheral blood of the two patients and four normal controls. *n* = 4; ∗∗∗*p* < 0.001; ns: no significant. *p* < 0.05 was considered statistically significant.

WGS revealed a large duplication with segmental heterogeneity. In genetic counseling, WGS was first performed on four affected individuals: the proband (F_II-6_), her daughter (F_III-6_), her younger sister (F_II-8_), and her nephew (F_III-7_). Consistent across all four patients, a 371 Kbp duplication was identified (seq[hg38] chr17: 59168097_59539137dup), encompassing two genes associated with autosomal-dominant RP: *GDPD1* and *YPEL2*. Notably, copy number analysis revealed segmental heterogeneity within the duplicated region. Three subsegments (A, B, C) were defined based on copy number repeat counts (Fig. 1D): segment A (length: 195 Kbp) with 3 copies; segment B (length: 21 Kbp) with 4 copies; segment C (length: 156 Kbp) with 3 copies. Validation against unaffected controls confirmed this dosage pattern. No additional pathogenic variants or RP-associated polymorphisms were identified in WGS data. Furthermore, WGS results from an unaffected individual (F_III-8_) revealed the absence of this duplication, demonstrating that the variation co-segregated within the family.

To address limitations of short-read next-generation sequencing in detecting large-scale rearrangements, we performed ONT on the proband (F_II-6_), yielding reads averaging ∼8 kbp. Leveraging its long-read capability, ONT enabled haplotype-resolved visualization of the duplicated region, confirming that the SV resided on a single haplotype (Fig. 1E). Copy number analysis revealed segment-specific dosage patterns: 2 copies for segments A and C, and 3 copies for segment B, consistent with WGS findings (Fig. 1E1). Breakpoint mapping identified two inversions flanking segment B (Fig. 1E2). Notably, only one read spanned the entire segment B (Fig. 1E2, red highlight), while other inversion-associated reads failed to cover both soft-clip regions simultaneously.

Figure 1F1 mapped key findings, including copy number alterations and breakpoint coordinates. Based on available evidence, we proposed multiple plausible configurations and listed three typical possibilities in Figure 1F2–1F4, including nested duplication-inversion, tandem triplication with interspersed inversions, and complex insertion. As the exact SV remained elusive, we employed OGM to resolve the precise architecture of the SV. The *de novo* assembly map confirmed the presence of two inversion breakpoints (Fig. 1G). The aberrant label pattern showed the comparative analysis between the proband and an unaffected control and supported the first hypothesized configuration (Fig. 1H1–1H3): a duplication-inversion involving segments A and B, followed by a duplication of segments B and C (Fig. 1F2).

Subsequent analysis of rearranged chromosome junctions near the two breakpoints using WGS data confirmed the microhomology-mediated end-joining (Fig. S1). Specifically, microhomologous sequences validated by Sanger sequencing were identified at the rearrangement junctions (Table S1), providing mechanistic insight into the duplication-inversion event. Corrected nucleotide positions were listed in Table S2, enabling complete resolution of this complex SV architecture. The SV identified in our study was characterized by copy number duplications of *GDPD1* and *SMG8*, alongside a *YPEL2* duplication harboring two intragenic inversions within introns 2 and 3. Accordingly, this rare variant has not been recorded in gnomAD, ClinVar, or the Decipher database, and no prior reports of it exist.

However, previous studies have identified eight other SVs spanning this genomic region in 22 autosomal-dominant RP families, which disrupted adjacent topological domains and up-regulated the expression of *GDPD1*, a gene encoding a retinal-specific enhancer.^5^ Notably, these SVs also differentially alter the expression of *YPEL2* and *SMG8* within this locus, thereby contributing to RP pathogenesis.^5^ Given the inaccessibility of ocular samples from affected individuals in our pedigree, we measured *GDPD1*, *YPEL2*, and *SMG8* mRNA expression in peripheral blood mononuclear cells as a surrogate marker of SV-mediated transcriptional perturbation, revealing prior findings. Relative mRNA expression levels of *GDPD1* and *SMG8* were significantly elevated in both F_II-6_ and F_III-6_ compared with the control, with no significant interpatient difference (Fig. 1I). Conversely, *YPEL2* mRNA levels were reduced in both F_II-6_ and F_III-6_ relative to the control, again with no significant interpatient difference (Fig. 1I). These results provide functional corroboration of SV pathogenicity, though the precise roles of *SMG8* and the most significantly deregulated gene, *GDPD1*, require further investigation.

Recently, SVs have attracted increasing attention. As the most widely used genetic testing method, next-generation sequencing can reveal mutational information associated with copy number variants in a subset of cases.^1^ In our study, WGS achieved satisfactory sequencing depth, consistently revealing large-scale copy number changes with segmental heterogeneity. The abundant SNV data generated by WGS also enabled cross-comparison with subsequent ONT results to precisely map SV breakpoints. Although next-generation sequencing-based WGS provided a solid foundation for diagnosing this complex SV, inherent limitations—its inability to resolve translocations or inversions—highlighted the necessity of integrating further methods.

ONT sequencing captures ultra-long genomic segments and enables haplotype phasing as well as simultaneous detection of translocations or inversions, providing critical insights into complex genome architecture.^3^ However, ONT also had limitations in our analysis. Specifically, insufficient read coverage spanning both soft-clip inversion breakpoints—likely due to the size of SV exceeding the average read length (∼8 Kbp)—hindered the exclusion of alternative mutational structures.

By phasing chromosome arms using ordered whole-genome maps of methyltransferase sites at the haplotype level, OGM enables visualization of complex SVs within continuous genomic mapping, overcoming limitations of traditional read-dependent approaches for assembling SV-containing regions.^4^ In our analysis, OGM is currently the most effective detection method for visualizing complex SVs—especially in cases where prior data suggest the presence of SVs and require confirmation of non-mirror-symmetric methyltransferase sites in this region.

Although gene expression analysis from peripheral blood mononuclear cells aligns with prior reports of disrupted adjacent topological domains driving retinal enhancer–gene interactions, it can only serve as supportive evidence rather than conclusive findings. The difficulty in obtaining retinal tissue for validations constitutes a major limitation of this study.

In summary, this integrative analysis combining WGS, ONT, and OGM resolved a complex SV in a large RP pedigree. By expanding the RP mutational spectrum and demonstrating the synergy of multiple approaches for SV characterization, this work advances genetic diagnostics and underscores the critical role of SV in inherited retinal diseases.

## CRediT authorship contribution statement

**Yuqiao Ju:** Writing – review & editing, Writing – original draft, Validation, Resources, Investigation. **Cong Duan:** Writing – original draft. **Kaixin Chen:** Validation, Resources, Investigation. **Qing Chang:** Funding acquisition. **Yuan Zong:** Writing – review & editing, Project administration, Funding acquisition, Conceptualization. **Fengjuan Gao:** Writing – review & editing, Project administration, Conceptualization. **Ge-zhi Xu:** Funding acquisition.

## Ethics declaration

The Institutional Review Board of Yunnan Eye Disease Clinical Medical Center, Affiliated Hospital of Yunnan University, Yunnan, China, approved information collection and analysis on October 19, 2022 (approval no. 2022205). Written informed consents were obtained from a three-generation pedigree, which was diagnosed with RP in November 2022, prior to ocular examinations and genetic tests. All study procedures conformed to the principles outlined in the Declaration of Helsinki.

## Data availability

All data and methods of genetic testing relevant to the study are included in the article or uploaded in Supplementary data.

## Funding

This work was supported by the 10.13039/501100001809National Natural Science Foundation of China (No. 82201204, 82171078) and the Shanghai Hospital Development Center Foundation (China) (No. SHDC12023116).

## Conflict of interests

The authors declared no conflicting interests.
