# Supplementary material for: Integrative optical genome mapping and long-read sequencing dissect a pathogenic complex structural variant in a multigenerational Chinese retinitis pigmentosa pedigree
Source: Genes Dis. 2025 Dec 23;13(6):102008. doi: 10.1016/j.gendis.2025.102008 (PMC13355571; doi:10.1016/j.gendis.2025.102008)
Supplement: Multimedia component 1 [file mmc1.docx]

**Supplementary Data**

**Methods and materials**

**1 Patients and clinical examinations**

A three-generation pedigree diagnosed with RP in November 2022 was enrolled in this study. The proband and all enrolled family members underwent comprehensive ophthalmic evaluations, including measurements of best-corrected visual acuity (BCVA, Snellen Chart), slit-lamp biomicroscopy (Keeler, Windsor, UK), intraocular pressure (NCT, Canon TX-20, Japan), and multimodal retinal imaging, including ultra-widefield fundus imaging and autofluorescence imaging (Optos UWF™, Scotland, UK), spectral-domain optical coherence tomography (SD-OCT, Heidelberg Engineering, Heidelberg, Germany), and full-field electroretinography (RetiMINER™, IRC, Chongqing, China). Axial length was measured using the IOL-Master 700 (Carl Zeiss, Germany). The diagnosis of RP was established by experienced ophthalmologists based on a comprehensive review of clinical manifestations, multimodal imaging findings, and electrophysiological assessments.

**2 Genetic testing**

***2.1 Whole genome sequencing (WGS)***

Peripheral blood samples from all participants were collected in EDTA-anticoagulant tubes, and genomic DNA was extracted using the FlexiGene DNA Kit (Qiagen, Hilden, Germany). Whole-genome sequencing (WGS) targeting over 23,000 genes was performed [1]. Library construction utilized the TWIST Library Preparation EF Kit 2.0 (104207), followed by sequencing on an Illumina platform (San Diego, CA, USA) to achieve an average coverage depth of ≥50×. All reads were aligned to the National Centre for Biotechnology Information (https://www.ncbi.nlm.nih.gov/) human reference genome (GRCh37/hg19) using Burrows-Wheeler Alignment (BWA) v0.7.17. Base calling and identification of SNP and Indel variants were conducted with GATK v4.2.0.0. Detected variants underwent functional annotation via Annovar, encompassing variant type, minimum allele frequency (MAF) in the general population (gnomAD database, http://gnomad-sg.org/), reporting status (HGMD database, https://www.hgmd.cf.ac.uk/ac/index.php), and disease association (OMIM database, https://omim.org/). Software tools (REVEL, dbscSNV, SpliceAI) predicted pathogenicity. Additionally, xhmm and clamms tools were employed to perform copy number variation (CNV) analysis on probe coverage regions. Variant analysis was conducted according to American College of Medical Genetics and Genomics (ACMG) guidelines [2].

***2.2 Long-read sequencing***

For long-read characterization, an Oxford Nanopore Technologies (ONT) protocol was employed, involving template preparation via multiplex long PCR, library construction with the SQK-LSK109 kit (ONT), and single-molecule sequencing [3].

The critical step was the preparation of sequencing templates via multiplex long PCR. Accordingly, primers were designed to amplify targeted sequencing templates, including the whole-gene sequence amplicons, as well as the DNA segment within SVs and CNVs detected by WGS. Library construction was conducted using the SQK-LSK109 kit (ONT). NEBNext® Ultra™ II End-Repair/dA-Tailing Module (NEB) was used for the end-repair of purified PCR product, according to the kit manual. After purification by Ampure XP beads (Beckman), each sample was barcode ligated with the A-tailed DNA via NEB and then eluted with nuclease-free water. The enriched long-length templates were diluted to 25 ng/μL and used as the sequencing library.

For Readfish, the host genome reference (Ensembl, GRCh38) index using minimap2 was first constructed. For UNCALLED, human cDNA and noncoding RNA (Ensembl, GRCh38) were used to build a BWA index as a reference to reduce the alignment time. Illumina raw reads were processed using Kneaddata (v0.7.4) to trim and filter low-quality sequences. Reads were aligned to the human reference genome (hg38) using minimap2 (v2.14-r883) using default parameters. Inversions were detected using npInv inversion caller. The coverage was assessed using mosdepth. The breakpoints and the junction of the structural rearrangements were identified manually by Integrative Genomic Viewer software.

***2.3 Optical genomic mapping (OGM)***

OGM and annotation of gDNA was performed at WeHealth Biomedical Technology (Shanghai, China).

**2.3.1 High Molecular Weight (HMW) DNA isolation and DNA labeling**

For DNA isolation, HMW genomic DNA was extracted by the Bionano Prep SP Blood and Cell Culture DNA Isolation Kit (Bionano Genomics, #80030) from fresh blood samples collected in EDTA-stabilized anticoagulative tubes. Briefly, we treated cells with lysis-and-binding buffer (LBB) to release gDNA that was bound to a nanobind disk, washed, and eluted in the provided elution buffer. The integrity and size of the isolated DNA were validated by pulsed-field gel electrophoresis. HMW DNA quantification was then performed with Qubit dsDNA assay BR kits using a Qubit 3.0 Fluorometer (Thermo Fisher Scientific). Unaffected control samples were obtained from healthy volunteers without RP phenotypes.

For DNA labeling, UHMW (Ultra-High Molecular Weight) gDNA molecules were labeled with the DLS (Direct Label and Stain) DNA Labeling Kit (Bionano Genomics, San Diego,CA, USA). We used Direct Label Enzyme (DLE-1) and DL-green fluorophores to label 750 ng of gDNA. After a wash-out of the DL-green fluorophores excess, DNA backbone was counterstained overnight before quantitation and visualization on a Saphyr instrument

**2.3.2 Data collection, genome assembly, and variant calling**

For data collection, fluorescently labeled DNA was pushed to the nanochannel array of a Saphyr Chip (Bionano Genomics), where the DNA linearization occurs. Then automated imaging of the linearized DNA was conducted by the Bionano instrument. DNA molecules were analyzed by generating **600** Gb of data per sample after 12–24h. The raw files from the Bionano Saphyr mapping platform in BNX formats were filtered based on a minimum length of 150 Kb and 9 labeled sites per single molecule. Areas with low individual assembly coverage (<92×, or 46× for chr Y) were defined as low coverage.

For genome assembly and variant calling, a set of label locations of a single DNA molecule was defined as a separate single-molecule map. We performed in silico DLE1 digestion of the human reference genome (GRCh38/hg38) to generate the reference map, and comparisons were performed using the software’s preset parameters. *De novo* assembly of single molecules to consensus genome maps was implemented with Bionano Solve v3.5.1. Analysis of the Bionano data was performed based on two separate pipelines: a CNV pipeline that serves to recover large unbalanced aberrations based on normalized coverage; and an SV pipeline that allows for detection of small SVs and comparisons between consensus genome maps and reference genome maps. The following filtering criteria were applied: hg38 DLE-1 SV mask was turned on to mask potential artifacts, common SV events, and highly repetitive regions of the genome such as segmental duplications.

The filter settings have been optimized with data from samples following prior analysis of OGM results to reduce the number of variants as follows. BED SV and CNV overlap precision were 12 kb and 500 kb respectively. The following confidence values were applied: insertion/deletion = 0, inversion = 0.7, duplications = -1, intra-translocation = 0.3, and inter-translocation =0.65, and CNV = 0.99. For CNV calls, only segments > 500 kb were considered. The minimal breakpoint region was defined by the boundary of the DLE mark location closest to the crossover points on each chromosome. Only SVs unreported from other population control samples were retained. For samples that showed negative results in the initial analysis, OGM data was re-analyzed with less stringent filter settings (lowering the CNV confidence value to 0.95 or turning off the hg38 DLE-1 SV mask.

**2.3.3 Data visualization** **and confirmation by Sanger sequencing**

For visualization, we used the Bionano Access software for genome mapping and manual examination of results. Circos plot and aberration details for individuals were constructed. The software was from https://bionanogenomics.com/support-page/ bionano-access/, a Bionano node.js web application. Aberrant novel SVs with breakpoints in centromeres, constitutive heterochromatin stretches, or p arm of acrocentric chromosomes were observed in some cases, but the detailed investigation into those was beyond the scope of this study, due to a lack of reference maps in those regions. The detected breakpoints were further validated by Sanger sequencing as previously reported[4]. Polymerase chain reaction (PCR) primers were designed using Primer3Plus (http://primer3plus.com/).

**3 mRNA Analysis of *GDPD1*, *YPEL2* and *SMG8***

To investigate whether structural variants affect gene transcription, relative gene expression levels were quantified using quantitative real-time PCR (qPCR). Total RNA was extracted from whole blood samples of two affected individuals and four healthy controls using the TIANGEN RNA extraction kit (Beijing, China). Reverse transcription was performed with the Hifair®-II-1st-Strand cDNA Synthesis Kit (Yeasen, Beijing, China), followed by qPCR amplification using SYBR Green PCR Master Mix (Novo-protein, Shanghai, China) on an RT-qPCR System (Roche, Shanghai, China). Primers targeting *GDPD1*, *YPEL2*, *SMG8* and the reference gene *ACTB* were designed using Primer3Plus 6.0 (http://primer3plus.com/cgi-bin/dev/primer3plus.cgi) and are listed in Supplementary Table 3. Relative mRNA expression was calculated using the 2^−ΔΔCt^ method, normalized to *ACTB*. All experiments were performed in quadruplicate. Statistical analysis was conducted via one-way ANOVA with Bonferroni post-hoc testing, with significance set at *p* < 0.05.

**References**

1. Kong, L., et al., *Mutations in VWA8 cause autosomal-dominant retinitis pigmentosa via aberrant mitophagy activation.* J Med Genet, 2023. **60**(10): p. 939-950.

2. Richards, S., et al., *Standards and guidelines for the interpretation of sequence variants: a joint consensus recommendation of the American College of Medical Genetics and Genomics and the Association for Molecular Pathology.* Genet Med, 2015. **17**(5): p. 405-24.

3. Jia, P., et al., *Haplotype-resolved assemblies and variant benchmark of a Chinese Quartet.* Genome Biol, 2023. **24**(1): p. 277.

4. Ju, Y., et al., *Genetic Characteristics and Clinical Manifestations of Foveal Hypoplasia in Familial Exudative Vitreoretinopathy.* Am J Ophthalmol, 2024. **262**: p. 73-85.

**Supplementary Figure 1. Preoperative and postoperative ultra-widefield fundus photographs of Cases 1–5.**


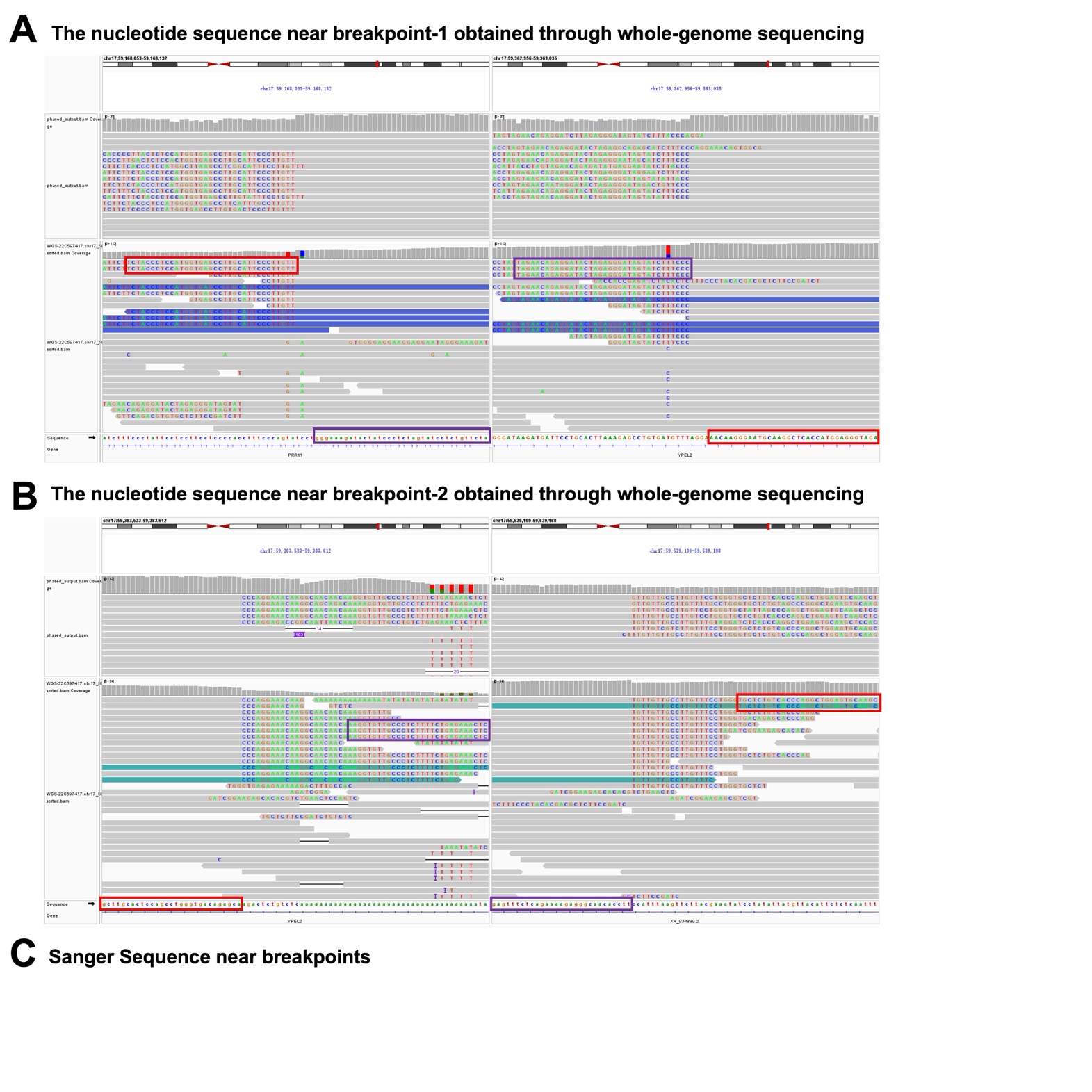


**Supplementary Table 1. The connection modes of derivative chromosomes at the breakpoints**

| **Joint Position** | | **Upstream Reference Sequence** | **Insert / Microhomologous Sequence** | **Downstream**  **Reference Sequence** |
| --- | --- | --- | --- | --- |
| Breakpoint  -1 | Chr17:59168097 | ACTAGAGGGATAGTATCTTTCCC | AGGA | TACTGGGAAAGGTGGGGAGGAAG |
|  | Chr17:59362996 | CACTTAAAGAGCCTGTGATGTTT |  | AACAAGGGAATGCAAGGCTCACC |
| Breakpoint  -2 | Chr17:59539162 | CTCAGAAAAGAGGGCAACACCTT | TGTTGTTGCCTTGTTTCCTGGG | CCATTTAAGTTCTTACGAAATAT |
|  | Chr17:59383565 | TTTTTTTTTTTGAGACAGAGTCT |  | TGCTCTGTCACCCAGGCTGGAGT |

**Supplementary Table 2. The corrected nucleotide position of the structural variant.**

| **Name of Segment** | **Region** | **Length (bp)** |
| --- | --- | --- |
| Upstream | chr17: - 59168096 | 168079 |
| A | chr17: 59168097 - 59362996 | 194218 |
| B | chr17: 59362997 - 59383561 | 21265 |
| C | chr17: 59383562 - 59539137 | 155576 |
| Downstream | chr17: 59539138 - | 160863 |

**Supplementary Table 3. Primers used for Quantitative Real-Time PCR**

| **Primer name** | **Sequence** | **Product length (bp)** |
| --- | --- | --- |
| *GDPD1*-F | ATTTGGAGAATACAATGGCAG | 182 |
| *GDPD1*-R | TTCATCTTTTGTGATATGGCA |  |
| *YPEL2*-F | CAAGGACAATGGCTGGGACTGAT | 201 |
| *YPEL2*-R | GCTTGGGAAGAATGTTCGGTTGA |  |
| *SMG8*-F | TGGAAGCTAAACTGCCGACCTTG | 279 |
| *SMG8*-R | ACCTTGAGGGCTCCATTGAGTTG |  |
| *ACTB*-F | AAGGTGACAGCAGTCGGTT | 192 |
| *ACTB*-R | TGTGTGGACTTGGGAGAGG |  |
